# Pediatric Heart Failure Pharmacotherapy: Transformative Insights for the Future

**DOI:** 10.31083/RCM44109

**Published:** 2025-11-25

**Authors:** Bibhuti B Das

**Affiliations:** ^1^Pediatric Cardiology, Methodist Children's Hospital, San Antonio, TX 78229, USA

**Keywords:** heart failure, children, HFrEF, HFpEF

## Abstract

This review aims to summarize the status and future directions of pediatric heart failure (HF) pharmacotherapy. Notably, managing HF in children presents unique challenges due to heterogeneous etiologies and a longstanding paucity of pediatric-specific data. While historically reliant on adult-derived evidence, current treatment strategies are evolving through an integration of novel and pediatric-focused therapies. Indeed, present pediatric HF algorithms, adapted from adult guidelines, now include four pharmacologic pillars: angiotensin-converting enzyme (ACE) inhibitors/angiotensin receptor blockers/angiotensin receptor–neprilysin inhibitors (ARNIs), β-blockers, mineralocorticoid receptor antagonists, and sodium–glucose cotransporter-2 (SGLT2) inhibitors. Multicenter registries, such as the Pediatric HF Registry, the Pediatric Cardiomyopathy Registry (PCMR), and the Advanced Cardiac Therapies Improving Outcomes Network (ACTION) HF medication titration projects, are further shaping a more evidence-informed and personalized approach. A comprehensive literature search was conducted using PubMed, Scopus, and Google Scholar to identify recent review articles, clinical trials, and guideline documents relevant to pediatric HF pharmacotherapy. The search focused on articles published in the English language from the past decade, with particular attention to transformative therapeutic insights. Data from adult HF studies were also included to provide context and bridge gaps in pediatric evidence. Where available, pediatric-specific data were prioritized to inform applicability. Relevant findings were critically appraised, synthesized, and integrated to develop a cohesive narrative reflecting current trends and emerging directions in pharmacological management of pediatric HF. This review examined the evolving landscape of medical therapies for chronic pediatric HF, underscoring the limitations of a one-size-fits-all approach. The heterogeneity of underlying etiologies complicates the development of guideline-directed treatments tailored to children, particularly when attempting to stratify care by phenotypes such as heart failure with reduced ejection fraction (HFrEF) and preserved ejection fraction (HFpEF), as is commonly practiced in adult populations. There is an urgent need to individualize treatment strategies based on the hemodynamic profile of each pediatric patient, advocating for the integration of precision-based care into guideline-directed medical therapy. Such an approach not only enhances clinical outcomes in a population marked by etiologic diversity and developmental variability but also informs scalable care models and future guideline frameworks that reflect the unique needs of children with HF.

## 1. Introduction

The International Society for Heart and Lung Transplantation defines pediatric heart failure (HF) as “a clinical and pathophysiologic syndrome that results from ventricular dysfunction, volume, or pressure overload, alone or in combination. In children, it leads to characteristic signs and symptoms, such as poor growth, feeding difficulties, respiratory distress, exercise intolerance, and fatigue, and is associated with circulatory, neurohormonal, and molecular abnormalities” [1]. HF imposes a major global health challenge, impacting individuals of all ages, including approximately 11,000–14,000 children hospitalized annually for HF in the United States alone [2]. Despite major advances in the diagnosis and management of adult HF, progress in pediatric HF has been comparatively limited due to the rarity of cases, the heterogeneity of etiologies, and the historical paucity of pediatric-specific clinical trials. Pediatric HF etiologies are broadly categorized into cardiomyopathies, congenital heart disease (CHD), and acquired conditions such as myocarditis or chemotherapy-induced cardiotoxicity. The unique challenges of pediatric HF, such as heterogeneous etiologies and the absence of large-scale randomized controlled trials (RCTs), have been detailed in a prior publication by the author [3]. Dilated cardiomyopathy (DCM) is the most common cause of HF in older children, while CHD-related HF dominates in infants. CHD-associated HF can be further classified into specific anatomical and physiologic substrates, including systemic left ventricular (LV) failure in bi-ventricular anatomy, systemic right ventricular (RV) failure (e.g., in patients with congenitally corrected transposition of the great arteries or following atrial switch repair), sub-pulmonary RV dysfunction (e.g., severe pulmonary valve regurgitation in repaired tetralogy of Fallot), and single-ventricle physiology with Fontan circulation, which poses unique long-term circulatory and multi-organ challenges.

The clinical presentation of pediatric HF is often age-dependent and may be subtle in infants, underscoring the need for a high index of suspicion. A thorough and structured diagnostic workup—guided by the 2014 International Society for Heart and Lung Transplantation (ISHLT) pediatric HF guidelines—is essential for accurate diagnosis [1]. This includes history and physical examination, laboratory biomarkers such as B-type natriuretic peptide (BNP) and N-terminal pro-B-type natriuretic peptide (NT-proBNP), electrocardiography, chest radiography, and echocardiography. Additional modalities like cardiac Magnetic Resonance Imaging (MRI), genetic testing, and cardiopulmonary exercise testing are increasingly used, especially in chronic or progressive cases. This review explores the evolving therapeutic landscape of chronic pediatric HF, highlighting recent pharmacologic advances, their mechanistic underpinnings, and implications for future practice and clinical research. In this review, pediatric chronic HF is examined in the context of the universal classification used in adults. Pharmacotherapy for pediatric HF is discussed based on left ventricular ejection fraction (LVEF), distinguishing between heart failure with reduced ejection fraction (HFrEF) and preserved ejection fraction (HFpEF).

## 2. Current State of Pharmacotherapy in Pediatric HFrEF

Historically, the management of pediatric HF has relied heavily on extrapolation from adult clinical trials due to the relative paucity of robust, pediatric-specific data. Landmark adult studies demonstrated the efficacy of angiotensin receptor–neprilysin inhibitors (ARNIs) and sodium-glucose cotransporter-2 inhibitors (SGLT2is), respectively, leading to Class I guideline recommendations for adults with HFrEF [4]. Over the past ten years, significant progress has been made due to legislation promoting pediatric-specific labeling for new market entries, alongside the establishment of collaborative, multicenter clinical trials [5]. In response, pediatric research has gained momentum, most notably with the Prospective Trial to Assess the Angiotensin Receptor Blocker Neprilysin Inhibitor LCZ696 Versus Angiotensin-Converting Enzyme Inhibitor for the Medical Treatment of Pediatric HF (PANORAMA-HF) trial, which evaluated sacubitril/valsartan in children with symptomatic HFrEF [6]. The study showed a significant reduction in NT-proBNP levels at 12 weeks, supporting its efficacy and safety, and resulting in U.S. Food and Drug Administration (FDA) approval for use in pediatric patients aged ≥1 year in 2019. This approval marked a pivotal shift, encouraging broader consideration of guideline-directed medical therapy (GDMT) in children. Since then, there has been progressive expansion in pediatric labeling and off-label use of existing HF therapies. Currently, the four foundational classes of HF medications used in adults- angiotensin-converting enzyme inhibitors (ACEi)/angiotensin receptor blockers (ARBs)/ARNIs, β-blockers (BBs), mineralocorticoid receptor antagonists (MRAs), and sodium-glucose cotransporter 2 (SGLT2) inhibitors—are increasingly incorporated into pediatric HF management. Carvedilol, for example, has been studied in the Pediatric Carvedilol Trial [7], although it did not meet its primary endpoint. Post hoc analyses suggested benefits in select subgroups, such as those with systemic LV. Similarly, enalapril and spironolactone have long-standing use in pediatric patients, particularly in congenital and DCM contexts [1]. Ongoing registry initiatives—especially the ACTION Pediatric HF Registry (capturing inpatient decompensated HF across 13–16 U.S. centers) and the NHLBI‑funded Pediatric Cardiomyopathy Registry (PCMR)—are actively contributing to a more evidence-based pediatric approach, tracking medication usage, hospitalization outcomes, ventricular assist device (VAD)/transplant rates, and longitudinal cardiomyopathy data.

### 2.1 Angiotensin Receptor-Neprilysin Inhibitors

**Fig. 1.**
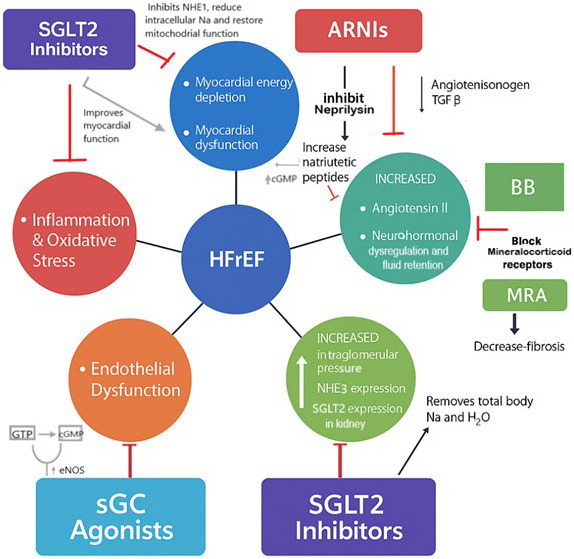
**Mechanisms of action of sodium-glucose cotransporter-2 inhibitors (SGLT2is), angiotensin receptor-neprilysin inhibitors (ARNIs), and soluble guanylate cyclase (sGC) agonist in the modulation of heart failure with reduced ejection fraction (HFrEF)**. (Original diagram). [NHE1, sodium/hydrogen exchanger isoform 1; NHE3, sodium/hydrogen exchanger isoform 3; cGMP, cyclic guanosine monophosphate; eNOS, endothelial nitric oxide synthetase; SGLT2is, sodium-glucose cotransporter-2 inhibitors; ARNIs, angiotensin receptor-neprilysin inhibitors; Na, sodium; AT_1_R, angiotensin receptor 1; MRA, Mineralocorticoid receptor antagonist; BB, Beta blocker; H_2_O, water; TGFβ, Transforming growth factor beta].

Sacubitril/valsartan is the first agent in a novel pharmacologic class known as ARNIs [8]. It combines two complementary mechanisms: sacubitril, a neprilysin inhibitor, and valsartan, an ARB. Sacubitril, administered as a prodrug, inhibits neprilysin—an endopeptidase responsible for degrading vasoactive peptides such as natriuretic peptides, bradykinin, adrenomedullin, and substance P. Inhibition of neprilysin leads to elevated levels of these peptides, promoting natriuresis, diuresis, vasodilation, and inhibition of pathologic hypertrophy and fibrosis, all of which are beneficial in reverse remodeling of the myocardium in HF [9]. However, neprilysin also degrades angiotensin II partially, and its inhibition alone can paradoxically increase angiotensin II levels, potentially worsening HF. To mitigate this, valsartan is co-administered to block the angiotensin II type 1 receptor (AT₁R), thereby preventing vasoconstriction, sodium retention, and aldosterone-mediated fibrosis. The synergistic effect of this dual blockade addresses two central pathways in HF pathophysiology: the renin-angiotensin-aldosterone system (RAAS) and the natriuretic peptide system (Fig. 1). Beyond hemodynamic effects, ARNIs have shown superiority over ACE inhibitors and ARBs in promoting reverse remodeling, improving ventricular function, and reducing cardiomyocyte apoptosis and extracellular matrix remodeling. These benefits are attributed to its unique ability to preserve endogenous protective peptides while simultaneously attenuating maladaptive RAAS signaling. Mechanistically, sacubitril/valsartan also modulates G-protein–coupled receptor pathways and may influence fibrosis and inflammation at the molecular level. Importantly, the therapeutic benefit arises only when sacubitril and valsartan are used in combination; monotherapy with either component does not yield the same magnitude of clinical or biochemical improvement among adults [10]. In the PANORAMA-HF trial, ARNIs did not demonstrate superiority over enalapril in treating pediatric HF due to systemic LV systolic dysfunction, based on a global rank endpoint at 52 weeks [6]. Nonetheless, both therapies yielded clinically meaningful improvements in functional status and NT-proBNP levels, with comparable safety profiles. As the pediatric evidence base continues to grow, ARNIs therapy is increasingly recognized as a cornerstone of GDMT for children with HFrEF, aligning pediatric practice more closely with adult HF management standards. In a propensity score–matched retrospective cohort study of 1038 pediatric HF patients from the TriNetX database, sacubitril-valsartan was not associated with a reduction in 1-year all-cause mortality or heart transplantation compared to ACE/ARB therapy (13.3% vs 13.2%, *p* = 0.95). While hypotension was more frequent with sacubitril-valsartan (10% vs 5.2%, *p* = 0.006), a trend toward fewer hospitalizations per year was observed [11].

### 2.2 Sodium-Glucose Cotransporter-2 Inhibitors

SGLT2is, also known as gliflozins, are a novel class of antihyperglycemic agents initially developed for the management of type 2 diabetes mellitus. These agents act by inhibiting the SGLT2 protein in the renal proximal convoluted tubule, which is responsible for reabsorbing approximately 90% of filtered glucose. By blocking this transporter, SGLT2is promote glycosuria and reduce blood glucose levels independently of insulin, while also exerting natriuretic and diuretic effects. Beyond glycemic control, SGLT2is have demonstrated robust cardiovascular benefits. These benefits are thought to arise from a combination of mechanisms, including reduction in preload and afterload, improved renal hemodynamics, reduced interstitial myocardial fibrosis, and enhanced myocardial energy metabolism through increased ketone utilization and mitochondrial efficiency (Fig. 1) [12]. Importantly, these effects occur regardless of diabetic status, as demonstrated in large placebo-controlled trials such as EMPEROR-Reduced [13].

While pediatric data remain limited, emerging case series suggest that SGLT2is are safe and potentially effective in children with HF. In a single-center retrospective study by Newland *et al*. [14], dapagliflozin was administered to 38 pediatric patients with HF, the majority of whom had dilated cardiomyopathy (68.4%) and LVEF ≤40% (65.8%). Patients received dapagliflozin alongside standard HF therapies, including ARNIs, BBs, MRAs, and diuretics. Over a median follow-up of 130 days, BNP levels significantly decreased from a median of 222 to 166 pg/mL, suggesting improvement in HF status. The medication was well tolerated, with no major changes in serum chemistries or vital signs. Importantly, there were no cases of hypoglycemia or hypovolemia, though 15.8% of patients experienced symptomatic urinary tract infections requiring antibiotics. In a subgroup of 16 patients with DCM, LVEF improved from 32% to 37.2% over a median follow-up of 313 days, indicating potential reverse remodeling. A second study by Konduri *et al*. [15] evaluated the use of SGLT2is in 14 adolescents (median age 14.5 years), including those with Fontan-associated heart failure, both with preserved and reduced EF. After an average of 4 months of therapy, reductions in BNP levels were observed without major adverse effects [15]. There were no reported incidents of genitourinary infections, hypoglycemia, diabetic ketoacidosis, or hypotension. One patient experienced significant diuresis and transient acute kidney injury, which was resolved with supportive care. These early findings suggest that SGLT2is may offer a promising therapeutic option in pediatric HF, including in complex cases such as Fontan physiology, where treatment options are limited. However, prospective trials are needed to establish safety, optimal dosing, and long-term efficacy in this population. In a recent study, the addition of dapagliflozin in 14 children significantly improved LVEF and symptoms, while demonstrating a favorable safety profile [16].

### 2.3 Hyperpolarization-Activated Cyclic Nucleotide-Gated Channel Blocker (Ivabradine)

Ivabradine is a heart rate-lowering agent approved for use in patients with HFrEF who are in normal sinus rhythm and have a resting heart rate ≥70 bpm despite maximally tolerated BB therapy. It selectively inhibits the funny current (If) in the sinoatrial node, reducing the influx of Na^+^ and K^+^ ions during diastolic depolarization. This slows pacemaker activity, prolongs diastolic filling time, and decreases myocardial oxygen consumption [17]. Elevated resting heart rates are associated with adverse LV remodeling, impaired diastolic filling, and worse clinical outcomes in HF. A systematic review of nine randomized controlled trials including 18,972 adult HF patients demonstrated that ivabradine significantly reduced HF-related mortality (RR 0.79) and hospitalization rates (RR 0.80) compared to placebo [17]. Unlike BBs or RAAS inhibitors, Ivabradine does not act on the neurohormonal system and thus has no effect on blood pressure or myocardial contractility, making it a more selective treatment option for certain HF patients. However, ivabradine was associated with an increased incidence of bradycardia, both symptomatic and asymptomatic.

Based on large-scale clinical trials such as SHIFT and BEAUTIFUL [18, 19], the American Heart Association (AHA) and European Society of Cardiology (ESC) guidelines recommend ivabradine for symptomatic HFrEF patients (NYHA class II–IV) with LVEF ≤35% and heart rate ≥70 bpm despite optimal treatment with BBs, ACE inhibitors (ACEis) or ARBs, and MRAs [4, 20]. In the pediatric population, ivabradine has shown encouraging results in improving HF outcomes. A randomized clinical trial in children with symptomatic chronic HF demonstrated significant reductions in heart rate and improvements in LVEF [21]. Based on these findings, the U.S. FDA approved ivabradine for use in children ≥6 months of age with symptomatic chronic HF and persistent tachycardia despite optimized BB therapy. Dosing regimens for ivabradine in children have been established by age and weight categories, ensuring safe titration. According to the 2014 ISHLT pediatric HF guidelines, ivabradine is considered reasonable (Class IIa, Level of Evidence B) for use as an adjunct therapy in children with stable HF when additional heart rate reduction is desirable [1]. More than 250 children across 19 pediatric HF centers have been treated with SGLT2 inhibitors, according to data gathered by the ACTION network. Preliminary results suggest the treatment is well tolerated, with no significant side effects reported during early follow-up.

### 2.4 Combination of ARNIs and SGLT2is

The combination of ARNIs and SGLT2is represents a promising therapeutic strategy in the treatment of chronic HFrEF. Clinical trials in adults have demonstrated that the concomitant use of these two agents provides synergistic benefits, leading to enhanced cardiovascular outcomes compared to either therapy alone [22]. This synergism stems from the convergence of complementary mechanisms targeting multiple pathophysiological pathways in HF (Fig. 1). Both ARNIs and SGLT2is contribute to preload reduction via natriuretic and osmotic diuresis, which decreases myocardial oxygen demand and improves cardiac output. Additionally, SGLT2is increase hematocrit by stimulating erythropoietin production, thereby enhancing oxygen delivery to peripheral tissues. At the cellular level, SGLT2is enhance myocardial energetics by promoting ketone body utilization, particularly beta-hydroxybutyrate, which serves as a more oxygen-efficient substrate compared to glucose or fatty acids. This metabolic shift leads to increased Adenosine Triphosphate (ATP) production and improved cardiac efficiency [12]. Moreover, elevated ketone levels act as endogenous histone deacetylase (HDAC) inhibitors, which reduce oxidative stress, inhibit cardiac hypertrophy, and attenuate inflammatory signaling through epigenetic modulation of peroxisome proliferator-activated receptors (PPARs) and pro-inflammatory cytokines [23, 24].

Myocardial fibrosis, a hallmark of adverse remodeling in HFrEF, is mitigated through transforming growth factor-beta (TGF-β) suppression, a pathway modulated by both ARNIs and SGLT2is therapy. ARNIs further contributes to antifibrotic effects by enhancing levels of natriuretic peptides, which promote anti-remodeling and vasodilatory signaling. Another critical target is the Na^+^/H^+^ exchanger isoform 1 (NHE1) and Ca^2+^/calmodulin-dependent protein kinase II (CaMKII), both of which are upregulated in HF and contribute to pathological calcium handling and mitochondrial dysfunction. SGLT2is directly inhibit NHE1 and suppress CaMKII activity, resulting in decreased intracellular sodium and reduced calcium overload, thereby preserving mitochondrial function and calcium homeostasis [25, 26].

Furthermore, SGLT2is enhance sarcoplasmic reticulum (SR) calcium cycling, which contributes to improved myocardial contractility and reduced arrhythmogenic potential [27]. These multifaceted cellular and molecular effects underscore the potential superiority of ARNIs–SGLT2is combination therapy as a next-generation cornerstone in the pharmacologic management of pediatric HFrEF.

### 2.5 Soluble Guanylate Cyclase (sGC) Stimulators

Vericiguat, a novel sGC stimulator, has gained increasing interest for its therapeutic potential in HF, particularly following encouraging results in adult clinical trials [28]. Unlike earlier agents, vericiguat has a dual mechanism of action: it enhances sGC sensitivity to suboptimal nitric oxide (NO) levels and directly stimulates sGC by binding to its regulatory heme domain even in the absence of NO. This dual action activates the NO–sGC–protein kinase G (PKG) signaling cascade, leading to an increase in cyclic guanosine monophosphate (cGMP) (Fig. 1), a key secondary messenger in cardiovascular homeostasis. In the failing heart, inflammation and endothelial dysfunction impair NO bioavailability, reduce sGC activity, and diminish cGMP levels. This dysregulation contributes to fibrosis, hypertrophy, and impaired myocardial relaxation [29]. sGC stimulators like vericiguat aim to restore this pathway, thereby attenuating adverse cardiac remodeling and improving hemodynamic function. In addition, when sGC is added to ARNIs and SGLT2is, there could be a more effective improvement of myocardial function and potential recovery of HFrEF.

Earlier-generation sGC stimulators, such as riociguat, were limited by short half-life and variable cGMP levels, which constrained their clinical utility in chronic HF. Vericiguat, developed through structural modifications to improve pharmacokinetics, demonstrated efficacy in the phase III VICTORIA trial, which enrolled high-risk adults with HFrEF (LVEF <45%) and recent worsening HF events [30]. Compared to placebo, vericiguat significantly reduced the composite endpoint of cardiovascular death or first HF hospitalization, earning it a Class IIb recommendation in recent adult HF guidelines [4, 20]. The drug was approved by the U.S. FDA in 2021 as an adjunct therapy for adults with symptomatic HFrEF. Despite these encouraging findings in adults, data in pediatric HF remain scarce. The ongoing VALOR-HF trial (Vericiguat in Pediatric Participants with Heart Failure Due to Systemic Left Ventricular Systolic Dysfunction) aims to bridge this gap [31]. This phase II trial evaluates the safety, efficacy, and pharmacokinetics of vericiguat in children with stable chronic HFrEF. The primary outcome is the change in NT-proBNP at 16 weeks, a surrogate marker for HF severity. Secondary outcomes include changes in log-transformed NT-proBNP at 52 weeks, time to first cardiovascular event (death, HF hospitalization, or worsening HF), adverse event profiles, and pharmacokinetics of tablet and oral suspension formulations.

Vericiguat’s unique pharmacodynamic profile—particularly its ability to function in states of low NO—makes it a compelling candidate for pediatric HF management, especially in advanced or treatment-resistant cases. If proven safe and effective, vericiguat could represent a valuable fifth pillar in the pharmacologic treatment of pediatric HFrEF. As the VALOR study progresses, collaboration among researchers, clinicians, and regulatory authorities will be pivotal to accelerate translation into pediatric practice and ultimately improve outcomes for children living with HF.

### 2.6 Omecamtiv Mecarbil (OM)

The hallmark of HFrEF is impaired myocardial contractility. While numerous agents have been developed to enhance contractile performance, traditional inotropes have been associated with increased arrhythmic risk, myocardial oxygen consumption, and mortality, limiting their long-term utility. OM represents a novel class of cardiac myosin activators, also known as myotropes, designed to increase contractility without raising intracellular calcium levels or oxygen demand [32]. OM selectively binds to the S1 domain of cardiac myosin, accelerating the rate-limiting step of ATP hydrolysis and strengthening the actin–myosin interaction. This mechanism prolongs systolic ejection time, enhances stroke volume, and improves overall cardiac output. Importantly, these effects are achieved without increasing myocardial oxygen consumption, distinguishing OM from classic inotropes. The Chronic Oral Study of Myosin Activation to Increase Contractility (COSMIC)-HF trial evaluated OM in adults with stable chronic HFrEF (EF ≤40%) [33]. Results demonstrated favorable remodeling, including prolonged systolic ejection time, increased stroke volume, and reduced LV end-systolic and end-diastolic volumes compared to placebo, suggesting improved contractile efficiency. However, physiologic improvements do not always translate into symptom relief. The Acute Treatment with Omecamtiv Mecarbil to Increase Contractility in Acute Heart Failure (ATOMIC-AHF) trial, which tested OM in adults hospitalized for acute decompensated HF (EF <40%), did not meet its primary endpoint of dyspnea relief, although trends toward hemodynamic benefit were noted [34]. The pivotal Global Approach to Lowering Adverse Cardiac Outcomes Through Improving Contractility (GALACTIC)-HF trial—a multicenter, randomized, double-blind, placebo-controlled phase III study—enrolled 8256 patients with symptomatic HFrEF (NYHA class II–IV, EF ≤35%) [35]. Participants had elevated natriuretic peptides and either a recent HF hospitalization or emergency department visit. The study found that OM significantly reduced the composite primary endpoint of time to first HF event or cardiovascular death (HR 0.92; 95% CI 0.86–0.99; *p* = 0.03). This benefit was primarily driven by fewer HF hospitalizations, with no significant difference in cardiovascular mortality (HR 1.01; 95% CI 0.92–1.11). Subgroup analyses revealed greater benefit in patients with lower EF and those in normal sinus rhythm. Notably, quality of life measures showed minimal overall change, although some improvement was observed in specific subpopulations (e.g., hospitalized patients). OM was associated with modestly increased troponin levels, but there was no excess in ischemic events, ventricular arrhythmias, or mortality compared to placebo, underscoring its favorable safety profile. While OM has not yet been studied in large-scale pediatric trials, its unique mechanism of enhancing contractility without increasing calcium influx or arrhythmogenic risk makes it a promising candidate for pediatric HF therapy. Further research-including pharmacokinetic, safety, and efficacy studies in children, is warranted to determine its potential role in pediatric HFrEF management.

## 3. Pharmacotherapy for HFpEF in Children

Pharmacologic treatment of HFpEF in children remains largely unsupported by robust clinical evidence, with current recommendations derived primarily from expert consensus and extrapolation from adult trials. Diuretics remain a cornerstone of symptomatic management in pediatric HFpEF, primarily used to relieve congestion, maintain urine output, and correct electrolyte imbalances [36]. Their administration requires careful titration, particularly in preload-dependent children with HFpEF, where excessive diuresis may lead to hypovolemia and compromised cardiac output. Importantly, there is no evidence that diuretics improve long-term outcomes, including mortality or hospital readmission rates, underscoring their role as supportive rather than prognostic therapy. Beyond diuretic therapy, the management of pediatric HFpEF emphasizes careful treatment of comorbidities such as systemic hypertension, arrhythmias, and obesity. Lifestyle interventions, including caloric restriction, structured aerobic and resistance exercise programs, and targeted weight management, remain underutilized but may improve functional capacity, endothelial function, and overall outcomes. Optimizing atrioventricular synchrony through rhythm control or pacing strategies is also crucial to preserving ventricular filling and stroke volume. Novel therapeutic approaches are emerging. SGLT2is have demonstrated morbidity and mortality benefits in adult HFpEF, and are under consideration in adolescents with advanced diastolic dysfunction, although pediatric-specific evidence is pending. Other agents with potential relevance include guanylate cyclase stimulators (e.g., vericiguat), myosin activators (omecamtiv mecarbil), and therapies targeting systemic inflammation and microvascular dysfunction—recognized contributors to HFpEF pathophysiology.

Spironolactone’s role in managing HFpEF is supported by findings from the Treatment of Preserved Cardiac Function Heart Failure with an Aldosterone Antagonist (TOPCAT) trial, which demonstrated clinical benefit among participants from the Americas [37]. As a mineralocorticoid receptor antagonist, spironolactone has been shown to improve diastolic function, attenuate myocardial fibrosis, and lower blood pressure—though its antihypertensive effects alone do not fully account for its impact in HFpEF. Beyond hemodynamic modulation, spironolactone influences molecular pathways involved in apoptosis and extracellular matrix remodeling, suggesting a multifaceted mechanism of action in this patient population [38].

**Fig. 2.**
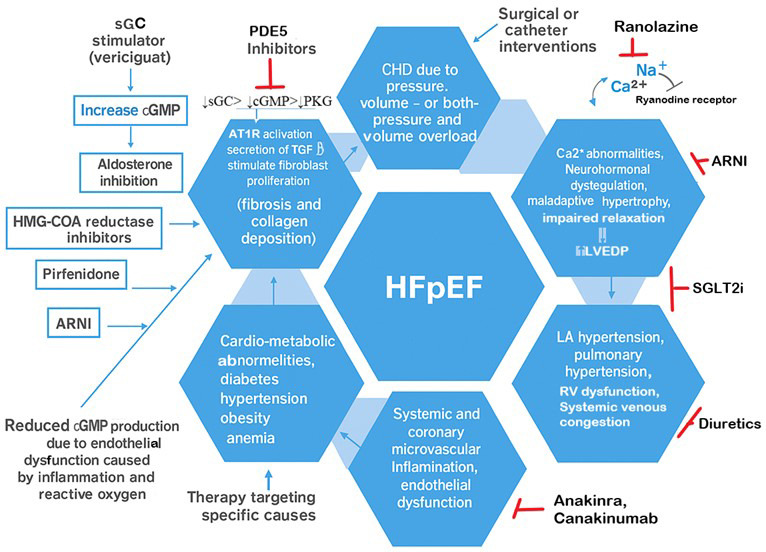
**Proposed mechanisms by which potential pharmacological agents modulate heart failure with preserved ejection fraction (HFpEF)**. (Original illustration). [cGMP, cyclic guanosine monophosphate; PHG, Protein kinase G; sGC, soluble guano cyclase; HFpEF, heart failure with preserved ejection fraction; ARNIs, angiotensin receptor-neprilysin inhibitors; SGLT2is, sodium-glucose cotransporter-2 inhibitors; HMG-CoA, 3-Hydroxy-3-Methylglutaryl-Coenzyme A].

Recent adult HF guidelines have upgraded SGLT2i to Class I, Level of Evidence A recommendation for the treatment of HFpEF [4, 20]. Although controlled pediatric studies are lacking, preliminary observational and uncontrolled data suggest the potential benefit of SGLT2is in children, including those with Fontan physiology or DCM patients [14, 15]. In light of this, SGLT2 inhibitors may be considered a Class IIb, Level of Evidence C option in pediatric HFpEF, especially in high-risk patients, while awaiting data from randomized pediatric trials to confirm their safety and efficacy [39]. SGLT2is, ARNIs, and sGC agonists target the pathophysiology of HFpEF—such as myocardial fibrosis, inflammation, and endothelial dysfunction, thereby improving cardiac output (Fig. 2).

**Fig. 3.**
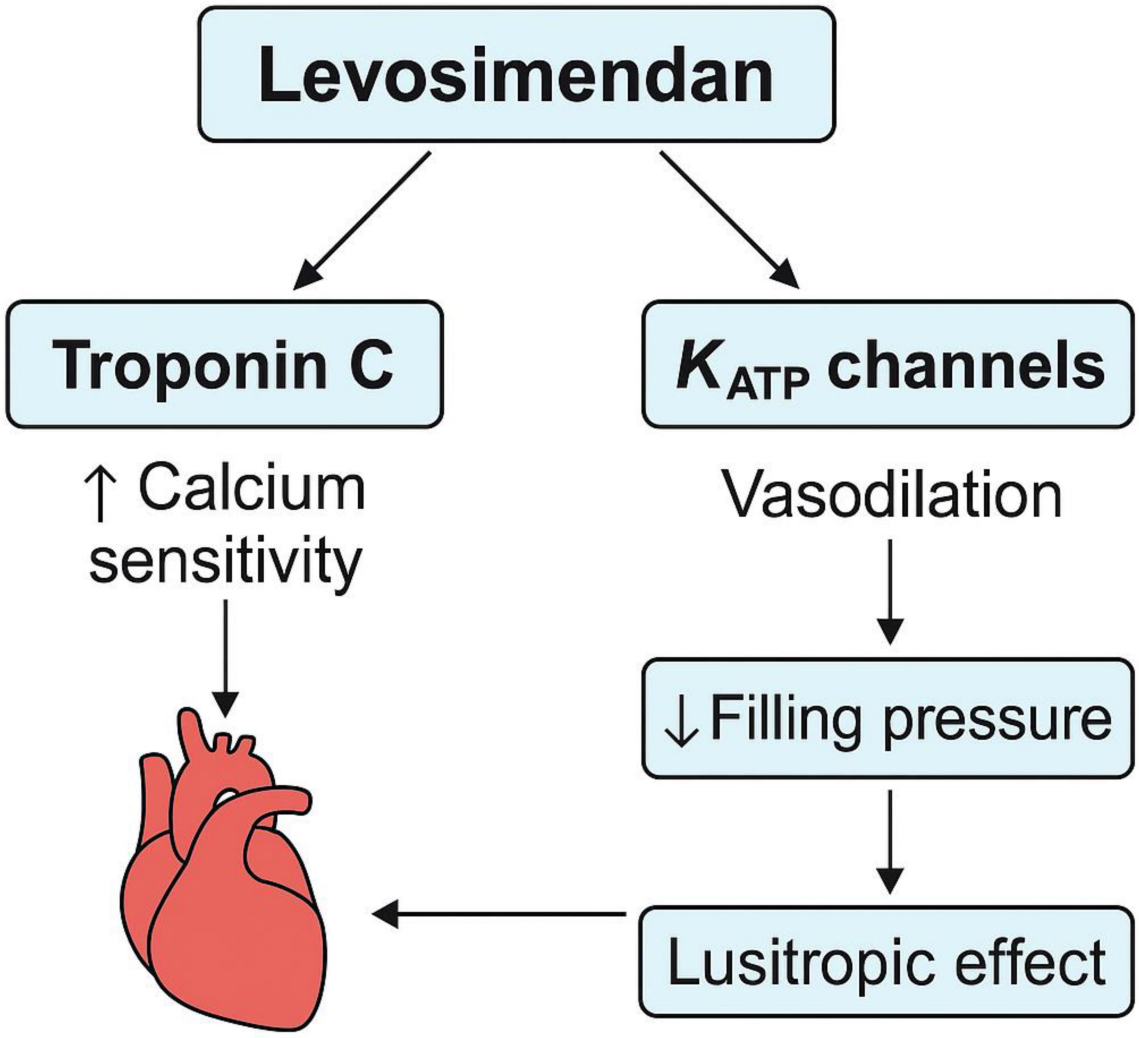
**Mechanisms of action of Levosimendan in addressing left ventricular diastolic dysfunction**.

Levosimendan is a calcium-sensitizing inodilator that enhances myocardial contractility without increasing intracellular calcium concentrations, thereby avoiding elevated myocardial oxygen demand. It binds selectively to cardiac troponin C in a calcium-dependent manner, enhancing the sensitivity of the contractile apparatus to calcium. This unique mechanism provides positive inotropic effects while preserving diastolic function [40]. In addition to its inotropic action, levosimendan opens ATP-sensitive potassium channels in vascular smooth muscle, resulting in vasodilation, reduced systemic and pulmonary vascular resistance, and decreased ventricular filling pressures (Fig. 3). These properties improve ventricular-arterial coupling and overall hemodynamics, including scenarios with HFpEF, where diastolic dysfunction predominates. Crucially, levosimendan has been shown to exert lusitropic effects—improving myocardial relaxation—likely through enhanced phosphorylation of phospholamban and improved sarcoplasmic reticulum calcium reuptake. These effects help optimize LV diastolic filling and compliance, key parameters in patients with elevated filling pressures and impaired ventricular relaxation. However, it is not available in the US.

Ranolazine, a partial inhibitor of fatty acid oxidation, promotes a metabolic shift in the heart toward glucose utilization, which consumes less oxygen than fatty acid metabolism. This shift may help sustain heart function during ischemic episodes. In adults, ranolazine has shown potential in managing diastolic dysfunction [41]. Studies in hypertensive mouse models have demonstrated reversal of diastolic impairment, possibly due to its direct influence on myofilaments [42]. Mechanistically, ranolazine acts by inhibiting the ryanodine receptor, reducing the late sodium current, and subsequently decreasing intracellular sodium and calcium concentrations during diastole, supporting better relaxation and compliance of the myocardium. In the RAnoLazIne (RALI)-diastolic HF clinical trial, intravenous administration in adults with HFpEF led to a slight reduction in left ventricular end-diastolic pressure (LVEDP) [43]. However, pediatric studies on ranolazine’s effects in HFpEF populations have yet to be conducted.

Finerenone, a novel nonsteroidal mineralocorticoid receptor antagonist, has been associated with a notable reduction in overall HF progression in adults. In the Finerenone in Heart Failure with Preserved Ejection Fraction trial (FINEARTS-HF), finerenone significantly reduced the composite rate of worsening HF events and cardiovascular death in adults with HF with minimal reduction in ejection fraction (HFmEF) compared to placebo [44]. While cardiovascular mortality alone was not significantly different, finerenone showed a favorable safety profile with reduced hypokalemia but increased hyperkalemia risk. However, studies in pediatric populations have not yet been conducted.

Emerging research using HFpEF models has highlighted a two-way relationship between metabolic strain and persistent inflammation, which appears to influence both systemic and cardiac immune activity involved in disease development. Elevated levels of inflammatory markers—such as interleukin-1, C-reactive protein, tumor necrosis factor-alpha, and soluble ST2—have been detected in individuals with HFpEF. Additionally, immune cells produce various pro-fibrotic mediators, including transforming growth factor-beta, interferon-gamma, Galectin-3, connective tissue growth factor, and angiotensin-converting enzyme, which collectively drive the transformation of fibroblasts into myofibroblasts and stimulate collagen buildup. While certain therapies like anti-inflammatory drugs (e.g., anakinra, canakinumab) have shown promise in adult HFpEF treatment [45]. There is currently no available data from pediatric populations.

Regardless of its underlying cause, 80% of adult patients with HFpEF develop pulmonary hypertension (PH), which typically begins as a passive consequence of increased LVEDP. However, in many cases, this evolves into pulmonary vascular disease (PVD), marked by vascular remodeling and vasoconstriction within the pulmonary venous, capillary, and arteriolar systems. These changes impair exercise tolerance, exacerbate pulmonary congestion, disrupt gas exchange, and are associated with increased mortality. A clinical trial is currently being conducted to explore the potential therapeutic efficacy and safety of sotatercept versus placebo in adults with PH due to HFpEF [46].

Currently, no RCTs have evaluated pharmacologic therapies for pediatric HFpEF associated with obesity or metabolic dysfunction. However, adult studies involving SGLT2 inhibitors, Glucagon-Like Peptide-1 Receptor Agonists (GLP-1RA) (e.g., semaglutide), and dual Glucose-dependent Insulinotropic Polypeptide (GIP)/GLP-1RA (e.g., tirzepatide) support pathway-directed strategies, particularly when HFpEF is driven by metabolic dysfunction [47, 48, 49, 50, 51]. While extrapolation from adult data may offer therapeutic insights, treatment decisions in children should be individualized, accounting for developmental physiology and clinical context.

## 4. Conclusions 

Recent advances underscore the urgent need for pediatric-specific pharmacology and trial design, emphasizing stratification by cardiac phenotype (e.g., CHD, systemic right ventricle, Fontan circulation, DCM), age-appropriate pharmacokinetic/pharmacodynamic considerations, and child-centered endpoints such as growth, biomarkers, and hospital-free days. Professional societies continue to highlight critical evidence gaps and advocate for mechanistically targeted, phenotype-aware investigations. Trials such as PANORAMA-HF demonstrated ARNIs-induced NT-proBNP reduction in children with HFrEF, though without superiority over enalapril at 52 weeks, reflecting the limitations of extrapolating adult data. Early registry findings suggest SGLT2 inhibitors may improve LVEF and functional status in children, but randomized trials are needed to confirm efficacy, safety, and dosing. In contrast, SHIFT-Peds offers more definitive support for ivabradine, showing heart rate reduction and improved LV function in symptomatic pediatric HFrEF. With growing investment in multicenter trials, refined biomarkers and imaging surrogates, and real-world registries like ACTION that capture clinical heterogeneity, the field is increasingly positioned to translate promising signals into durable, child-centered outcomes. Nonetheless, the distinct pathophysiology and pharmacology of pediatric HF demand deeper biological insight to guide precision therapeutics. Despite ongoing challenges, momentum is building—driven by innovation, collaboration, and a shared commitment to advancing personalized care and improving long-term outcomes for children with HF.

## Abbreviations

NYHA, New York Heart Association; ISHLT, International Society for Heart and Lung Transplantation; SGLT2is, sodium-glucose cotransporter-2 inhibitors; sGC stimulator, Soluble Guanylate Cyclase Stimulator; ACEI, angiotensin-converting enzyme inhibitors; ARB, angiotensin II receptor blockers; ARNIs, angiotensin receptor-neprilysin inhibitors; BNP, B-type natriuretic peptide; NT-proBNP, N-terminal pro-B-type natriuretic peptide; HFrEF, heart failure with reduced ejection fraction; HFpEF, heart failure with preserved ejection fraction; cGMP, cyclic guanosine monophosphate; GTP, guanosine triphosphate; PKG, protein kinase G; FDA, Food and Drug Administrator; ACTION, Advanced Cardiac Therapies Improving Outcomes Network; PANORAMA-HF, Prospective Trial to Assess the Angiotensin Receptor Blocker Neprilysin Inhibitor LCZ696 Versus Angiotensin-Converting Enzyme Inhibitor for the Medical Treatment of Pediatric HF.
